# Osteoimmunology: A Current Update of the Interplay Between Bone and the Immune System

**DOI:** 10.3389/fimmu.2020.00058

**Published:** 2020-01-31

**Authors:** Christian Guder, Sascha Gravius, Christof Burger, Dieter C. Wirtz, Frank A. Schildberg

**Affiliations:** ^1^Clinic for Orthopedics and Trauma Surgery, University Hospital Bonn, Bonn, Germany; ^2^Department of Orthopedics and Trauma Surgery, University Medical Center Mannheim of University Heidelberg, Mannheim, Germany

**Keywords:** osteoimmunology, bone, immune cells, orthopedics and trauma surgery, bone fracture, rheumatoid arthritis, osteoporosis, prosthesis failure

## Abstract

Immunology, already a discipline in its own right, has become a major part of many different medical fields. However, its relationship to orthopedics and trauma surgery has unfortunately, and perhaps unjustly, been developing rather slowly. Discoveries in recent years have emphasized the immense breadth of communication and connection between both systems and, importantly, the highly promising therapeutic opportunities. Recent discoveries of factors originally assigned to the immune system have now also been shown to have a significant impact on bone health and disease, which has greatly changed how we approach treatment of bone pathologies. In case of bone fracture, immune cells, especially macrophages, are present throughout the whole healing process, assure defense against pathogens and discharge a complex variety of effectors to regulate bone modeling. In rheumatoid arthritis and osteoporosis, the immune system contributes to the formation of the pathological and chronic conditions. Fascinatingly, prosthesis failure is not at all solely a mechanical problem of improper strain but works in conjunction with an active contribution of the immune system as a reaction to irritant debris from material wear. Unraveling conjoined mechanisms of the immune and osseous systems heralds therapeutic possibilities for ailments of both. Contemplation of the bone as merely an unchanging support pillar is outdated and obsolete. Instead it is mandatory that this highly diverse network be incorporated in our understanding of the immune system and hematopoiesis.

## Introduction

In 1972, pioneering studies were able to show the close relationship between the immune system and the bone, by identifying osteoclast-activating substances in immune cells (1, 2). Almost 30 years later, Arron and Choi coined the term “osteoimmunology” in a letter to *Nature*, recapping an article Takayanagi et al. wrote earlier that very year, about the importance of RANKL and IFN-γ secretion of activated T cells (3, 4).

Now, in 2019, the field of osteoimmunology is as important and thriving as ever, allowing for the unparalleled opportunity of understanding processes like arthritis and rheumatic diseases. Its conclusions have made it possible to treat AIDS patients with higher accuracy and improve osteoporotic patients' activities of daily living. To understand osteoimmunology one must identify the lowest common denominator of the immune system and bone, their conjoint heritage in stem cells, and the consequences in their shared signaling pathways. Today it is evident that both systems influence each other greatly and that the impact to human physiology and pathology is extensive.

This article aims to present an overview of the topic, illuminate possibilities of treating patients with certain diseases and pinpoint the fascinating future potential of osteoimmunology.

## Bone and Bone Cells

Osseous tissue roughly consists of two compartments: compacta and spongiosa. The compacta functions as mostly structural support for bodily stability and movement and as a pool for calcium if needed. It comprises the outermost layer of every bone and itself is composed out of osteons (5). The spongiosa is a trabecular, highly porous network of bone, housing red and white bone marrow, berth of hematopoiesis. It is highly vasculated, albeit not innervated like compacta (5). Both regions of osseous tissue rely on the activity of mainly three types of cells: Osteoclasts (OC), osteoblasts (OB), and osteocytes, with the latter making up about 95% of the total cell population (6).

### Osteoblasts

The osteoblast (OB) can be considered the anabolic part of the cell triad. OBs deposit recently synthesized extracellular matrix, called osteoid, consisting mainly of collagen type 1, proteoglycans, and water. Only after the osteoid is mineralized with hydroxyapatite crystals does it obtain the stability needed for normal workload (7). Detailed mechanisms of bone healing and remodeling will be discussed later in conjunction with osteoclast activity.

**Figure 1:**
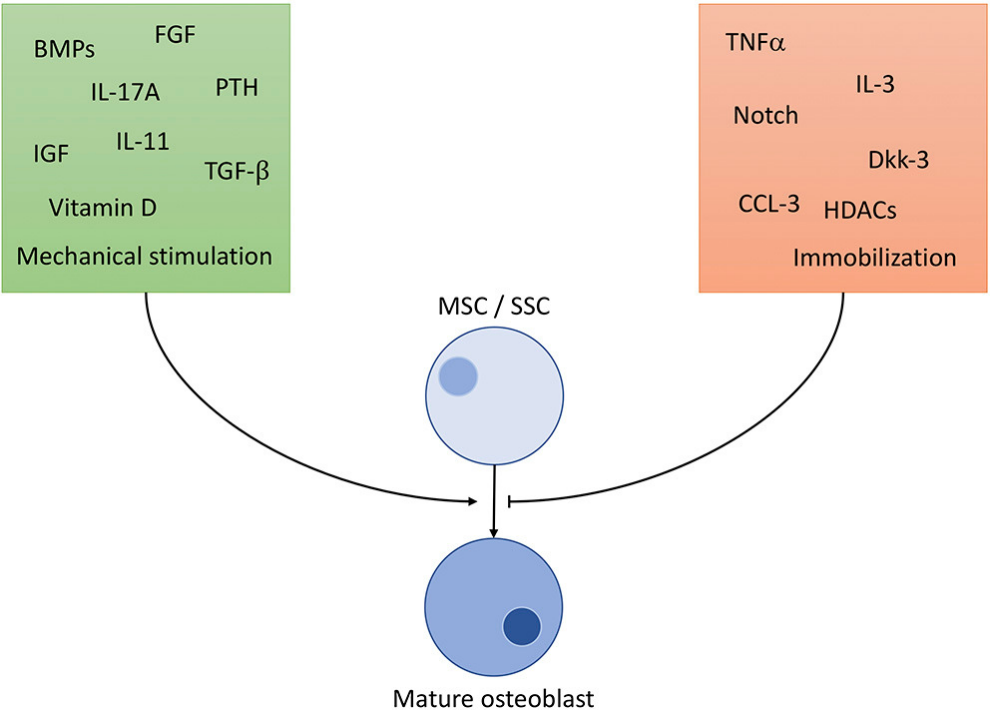
Selected important players of osteoblast differentiation. Promoting factors (green square) include: BMP: bone morphogenic proteins of the TGF-β superfamily, a large (>20) group of anabolic proteins secreted by different cells to ensure development of bone, cartilage and tendons mainly via the Runx2 axis. TGF-β: transforming growth factor β_1−3_, contained in bone cartilage tissue in large amounts. Regulates osteoblast differentiation and development similar to BMPs, with additional anti-inflammatory properties. IGF: insulin like growth factor, mainly promotes osteoblast activity, but also has positive effect on differentiation. FGF: fibroblast growth factor, stimulates OB differentiation in a manner similar to BMP signaling (Runx2 pathway). IL-11: is directly induced by mechanical strain in bone and PTH signaling and downregulates effects of Wnt inhibitors like Dkk-1/2. IL-17A: effects depend on targeted cell type. MSC differentiation is supported, but calvarial preosteoblasts receive negative effects. PTH: parathyroid hormone, only stimulates OB differentiation through Runx2 if elevation of PTH is intermittent, continuously elevated levels result in bone resorption. Vitamin D: complexes Runx2 and other cofactors to upregulate osteoblast specific genes. Mechanical stimulation promotes bone growth and OB activity according to Wolff's law: bone structure forms according to applied force. Inhibiting factors (orange square): TNFα: supposedly hampers with differentiation through inhibition of IGF-1, BMP-2/6 and possibly guiding SSCs to lineages other than osteoblasts. Dkk: the Dickkopf ligand family are potent inhibitors of the Wnt pathway, thus inhibiting development. HDCAs: histone deacetylases, prominently HDCA3-7 inhibit Runx2-DNA interaction, interfere in gene translation. CCL-3: also known as macrophage inflammatory protein 1-α (MIP-1-α) promotes inflammation and osteoclast activation. Additionally, it blocks the Runx2 pathway in OBs. Notch: effects of Notch on OBs are not fully clarified, although findings constitute a hampering function by Runx2 inhibition. Immobilization: absence of mechanical strain results in OB inhibition by non-triggering of Wolff's law.

OBs derive from skeletal stem cells (SSC), a subtype of the mesenchymal stem cell (MSC) line, situated in the bone marrow. SSCs can mature into different types of cells of the skeletal system, namely chondrocytes, adipocytes and OBs (8). To ensure commitment to the correct cell line, OB precursors are exposed to a highly specific cocktail of differentiation factors, partly portrayed in Figure 1 (9–18). The exact composition of factors is yet to be unraveled and understood (8). It is clear, however, that certain cells of the immune system contribute to the differentiation of OBs: It has been shown, that γδ T cells, a not antigen (AG)-specific subgroup of T cells, secrete IL-17A, formerly mainly associated with OC activation, to quicken OB proliferation and differentiation (19). Macrophages have massive influence on OB activity by emitting TNFα, being one of the most potent OB differentiation inhibitors (20). OBs are sensitive to PTH (parathyroid hormone), an anabolic hormone secreted by the parathyroid gland, which stimulates bone formation and triggers OB's secretion of pro-hematopoietic factors like IL-6, IL-6R, and MCP-1 (21).

The most important player in the development from SSC to osteoblast is the Wnt Pathway. The term Wnt comprises of a large group of signaling molecules that promote osteoblastogenesis and inhibit adipogenesis. Loss of function mutations within this pathway result in an osteoporotic phenotype, while gain of function mutations support (pathological) high bone mass (22). To support bone formation Wnt molecules activate G-protein coupled receptors (Fzd) and coreceptors of the Lrp family. Activation results in a signaling cascade, typically containing β-catenin, effectively upregulating aerobic glycolysis, β-oxidation and other anabolic mechanisms (23, 24) through activation of the Runx2 gene (25).

Other than Wnt, the BMP pathway (bone morphogenic protein) is able to upregulate OB activity and differentiation by activating Runx2 (25). After binding to the BMP receptor (BMP-R) the molecules cause dimerization of BMP-R and following phosphorylation of Smad proteins (26). After phosphorylation, those molecules then activate Runx2 (25). Despite its name, the BMP molecule family also plays important roles outside bone formation. BMP molecules influence embryogenesis and promote muscle and neuronal growth (27, 28), and are key players in the development and regulation of the immune system. T cell differentiation and activation is dependent on BMP signaling as well as Runx2 regulation (29). In B cells, BMP-6 inhibits lymphopoiesis (30) and general growth (31). In macrophages, too, proliferation is inhibited and pro-inflammatory pathways hampered (32, 33).

Downregulation of the Wnt pathway is controlled by antagonists, e.g., Dickkopf1/2 and Sclerostin, blocking the interaction between Lrp and Fzd (34). Sclerostin is a marker for mature osteocytes, since OBs themselves have not been observed to secrete sclerostin (35, 36), and capacity of production seems to increase with age (37). Sclerostin knockout mice (SOST^−/−^) show immensely increased osteoblastic activity resulting in smaller bone marrow cavities and subsequently impaired hematopoiesis, especially regarding B cells (38). These findings highlight the importance of regulated bone mass for hematopoiesis and, secondly, indirect influence of an osseous glycoprotein for cells of the lymphatic lineage. While mentioned findings emphasize the importance of conjoined signaling on a molecular level, these statements can be transferred to cell activity.

Studies have promoted relevance of OBs for the hematopoietic system. In 2003, it was pointed out that the OB number strongly associates to the number of hematopoietic stem cells (HSC), and furthermore ablation of OBs directly results in decrease of HSC number (39). OBs and members of the OB cell line secrete factors like N-cadherin, angiopoetin-1, thrombopoietin and osteopontin to regulate the size of the HSC pool and are able to control when HSCs migrate in and out of the bone marrow (40). Osteopontin, a member of the SIBLING glycoprotein group, is of particular interest for modern medicine, because it is overexpressed in patients suffering from certain types of cancer and correlates to metastasis and general aggressiveness (41). Additionally, OBs play an important role in lung adenocarcinomas. These tumors have been shown to have a positive effect on OB growth rate; conversely, OBs endow tumors with specific, SiglecF^high^ neutrophils, which promote tumor growth (42). Moreover, it has been shown that defects in hematopoietic niches can contribute to the development of leukemia (43), while activation of osteoblasts increased mouse survival and lowered symptoms of leukemia. Inversely, acute myeloid leukemia diminishes osteoblasts in humans (44).

While these findings present the importance of OB activity on HSC regulation, other studies deemphasize OB contribution. Biglycan-deficient mice show an osteoporotic phenotype and significant reduction in OB numbers, but no differences in hematopoiesis (45). In 2007, Lymperi et al. found that treating OBs with strontium, an anabolic, bone-stimulating factor, resulted in increased OB number and general bone volume, but not in increased HSC count (46). Further investigation on OB's influence on the HSC is obligatory to work out the role of OBs in leukemia and other related illnesses.

Terashima and Takayanagi have recently published a paper discussing the role of OBs in sepsis, i.e., systemic symptoms attributable to infection. While the early stages of sepsis are accompanied by an extreme inflammatory response, later stages are marked by immunosuppression. Both states result in high mortality rates; the latter is beneficiary to secondary- or superinfections. The main factor of immunosuppression is the depression of immune cells, primary B and T cells, and their progenitors for up to 28 days (47–49). The group makes the assumption, that the remarkable duration of immunosuppression stems from OB depletion, hindering HSC tending by OBs. OB depletion is in part caused by a significant increase of inflammatory factors like IL-1β, TNFα, and G-CSF during the inflammatory phase of sepsis (50), making OBs a possible target for sepsis treatment (51).

### Osteocytes

Osteocytes are not an independent type of cell line, but rather the last stage in OB development. The transformation begins passively by its “entrapment” in synthesized matrix, situating itself in so-called lacunae (52). The secluded position of the osteocytes makes it hard to signal for other cells and lengthens diffusion distances tremendously, thus dendrites are needed. Information on the transition remains scarce, although major factors have been identified. Aside from mechanical components (pressure, tension, low oxygen stress, matrix mineralization), FGF-2, oncostatin, and retinoic acid contribute to osteocyte differentiation (53).

Preosteocyte cell bodies undergo drastic changes as they develop dendrites to form contact with other osteogenic cells, via gap junctions (52). Although osteocytes have relatively slow metabolism, no ability for mitotic division and are anchored to their surroundings, they have great influence on bone metabolism. Not unlike the nervous system, they form contact points with each other, OBs and OCs, spanning a network over the entire bone. This way osteocytes are able to conduct bone turnover (53). To do so they recognize fluid shear stress, the movement of fluid in the duct system of the bone. Through mechanotransduction, these stimuli are translated into secretion of factors (54). Lack of mechanical strain will result in paracrine secretion of FGF-23, RANKL, and sclerostin. All of the aforementioned are inhibitors of bone growth. FGF-23 inhibits bone mineralization (55). RANKL is the most potent activator of OC differentiation and activation. To stimulate growth and mineralization, recognized mechanical strain will result in osteocytic secretion of OPG, a decoy receptor of RANKL, preventing its interaction with RANK (56).

Microgravity causes an impaired osteocyte network formation in mice, which subsequently reduces lymphocyte number in involved bone marrow, but not systematically. To prove this result was due to osteocyte impairment in microgravity and no other factors, osteocyte ablation (OL) was induced in “osteocyte-less (OL) mice.” Compared to wild type litter mates, OL mice exhibited a reduced osteocyte network and severe B and T cell lymphopenia (57). By depletion of GSα, a crucial osteocyte receptor, Fulzele et al. presented mice with dramatically increased myeloid stem cells in the bone marrow and overall system, indicating the importance of G-CSF secreted by osteocytes (58). Nonetheless, OL mice exhibit no change in myelopoiesis, hinting toward interchangeability of osteocytes regarding G-CSF production.

Impressively, atrophy of lymphatic organs (esp. spleen and thymus) can be observed in OL mice (57), raising the question whether osteocytes are important for the structural integrity of these organs and/or cultivation of relevant effector cells.

### Osteoclasts

Osteoclast function is the destruction and clearance of osseous tissue. This is not solely a catabolic task, since recently disintegrated matrix can only then be rebuilt by OBs. The assurance of resilient bone mass is therefore dependent on OBs as well as OCs (6).

OCs are large (50–100 nm), multinucleated cells originating from HSCs rather than mesenchymal stem/stromal cells (MSC) (59), constituting the proximity to macrophages. The lytically active, ruffled border on the bone matrix-facing side is heavily creased to increase the surface area. This membrane contains a high number of H^+^-ATPases, lowering the pH to around 4.5 to dissolve chemical bonds of calcium in the matrix. Around this active border OCs are affixed to the osseous tissue by integrins, to ensure a tight seal around the area of low pH. To disintegrate proteins, mainly collagen type I, OCs primarily secrete Cathepsin K amongst other Cathepsins and matrix metallopeptidases (60, 61). After lysis of organic and inorganic material OCs assimilate fragments via endocytosis (62).

**Figure 2:**
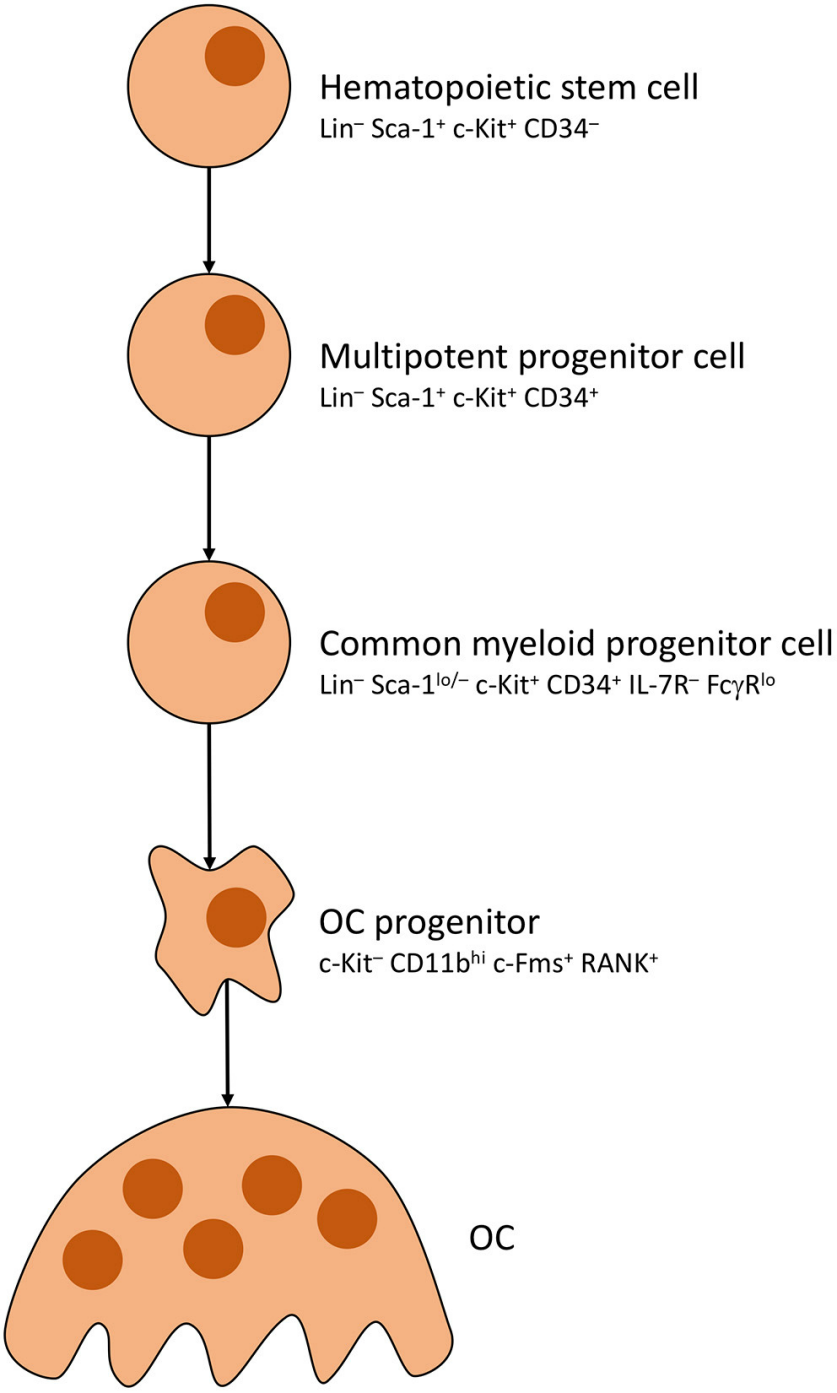
Osteoclast development. Simplified presentation of OC development. Other than OBs, OCs develop from hematopoietic stem cells. Stem cells lose self-renewing potential during development. Other blood cells branch off from multipotent progenitor cells. OC progenitor cells iterate to become RANK^+^, then fuse into OCs with multiple nuclei.

As mentioned before, OCs underlie strict control by, mainly, osteocytes. Activation is primarily steered by adjusting rates with which OCs develop from precursors (OCP). To do so, osteocytes distribute mesenchymal colony stimulating factor (M-CSF) to MSCs, which causes commitment to the OC cell line thus creating said precursors (59). OC development is pictured in Figure 2. Note that RANK positivity develops in the last stages of OCP development, shortly before multiple OCPs fuse into immature osteoclasts, which mature into full osteoclasts (63, 64). Most important factor for OCP fusion and maturation is Receptor Activator of NF-κB Ligand (RANKL) secreted by osteocytes (65).

**Figure 3:**
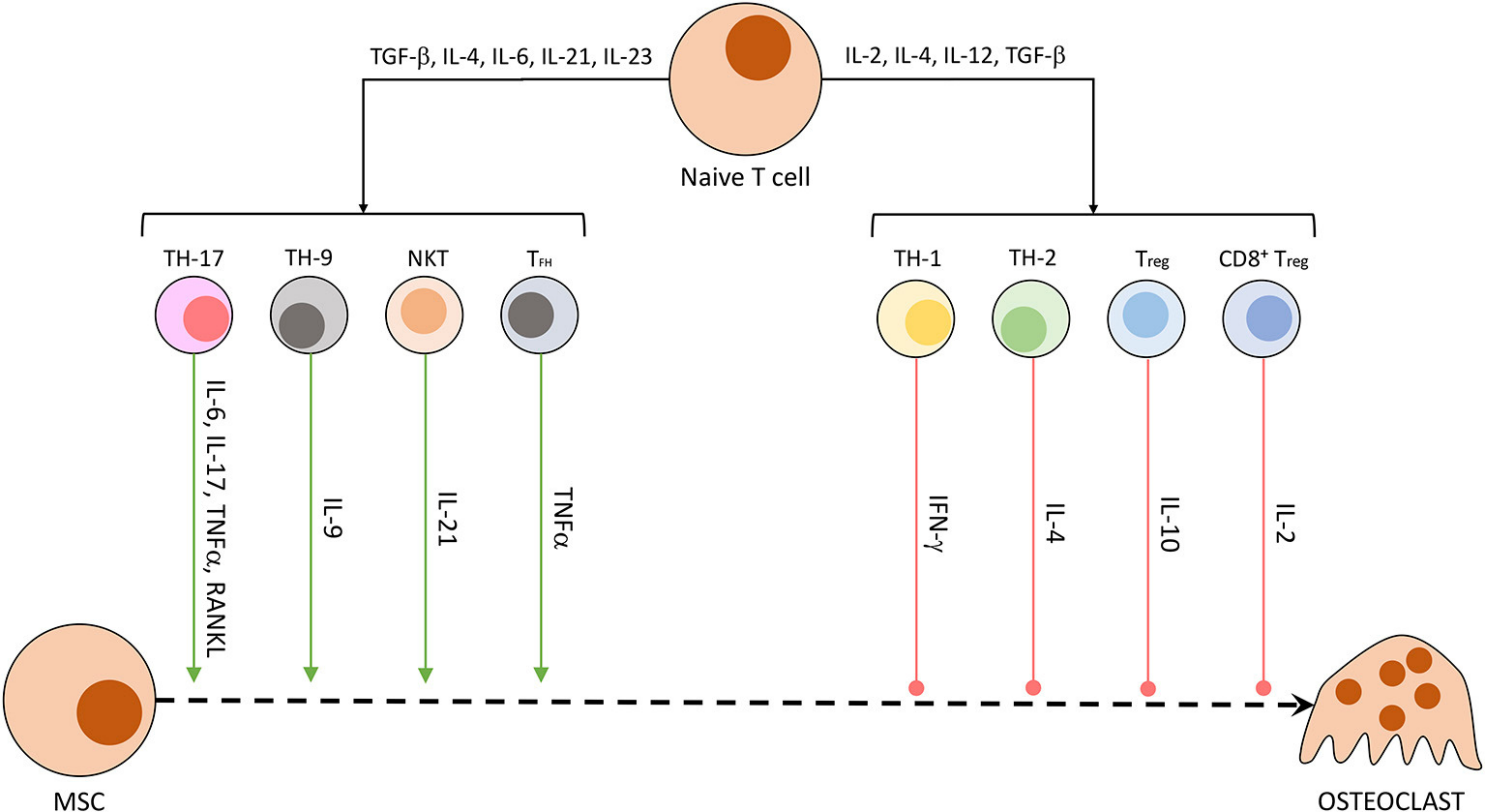
Secreted T cell factors modulating osteoclastogenesis. Naïve T cells undergo differentiation in dependency of exposed factors. A mix of pro-inflammatory and anti-inflammatory conditions leads to the development of differentiated T cell subtypes. These specialized cells then produce a characteristic fingerprint of soluble molecules, which induce or hamper OC development and activity.

Other than osteocytes, different cells of the mesenchymal cell line have been shown to secrete RANKL and other OC-stimulating factors. Especially synovial fibroblasts produce RANKL when triggered by IL-17, produced by T helper cells, and TNFα, originating from macrophages. B cells of the immune system directly stimulate OC precursors through the production of IgG antibodies and RANKL (40, 66). In patients suffering from rheumatoid arthritis or enthesitis-related arthritis, the synovia and surrounding tissue has been shown to contain a significantly higher amount of RANKL (67) and other pro-inflammatory cytokines (IL-17, IL-23, TNFα) (68). RANKL activates NFATc1 in a TRAF6-dependent pathway, which ultimately promotes OC-specific protein transcription and OC maturation. NFATc1 is a protein first discovered in T cells, subsequently named *nuclear factor of activated T cells, cytoplasmatic 1*. While NFATc1 gene and protein regulate maturation in OCs, in CD8^+^ T cells it controls the strength of cytotoxic reaction against targeted cells by IL-2 and IFN-γ (69). The influence of T cells on osteoclastogenesis is summarized in Figure 3 (40, 70). Th17 cells, Th9 cells, NKT cells, and follicular helper T (TFH) cells are promoting the process of osteoclastogenesis and thereby induce bone loss. Contrarily, Th1, Th2, T_reg_, and CD8^+^ T cells inhibit the generation of osteoclasts and therefore reduce bone loss (40, 70).

Whether RANKL is indispensable for OC development is subject to controversy. Multiple substances have been found to trigger osteoclastogenesis in absence of RANKL, like TNFα (71–73), APRIL (A proliferation-inducing ligand), IGF I+II (74), and TGF-β (75), to name a few. However, methods of OC extraction for most of these studies has been heavily criticized, because of their inability to select OC progenitors exclusively. Extracted cells include MSCs, which themselves are capable of RANKL production (64). New methods of highly specific OC progenitor extraction are needed to definitively prove or disprove RANKL independent osteoclastogenesis.

Similar to OBs, OCs have influence on hematopoiesis. A somewhat average bone density with cavities for hematopoiesis is required for a functioning immune system and regular erythrocyte numbers, proven by the fact that osteopetrosis, due to OC defects, can lead to anemia and infections (76, 77).

Studies found that introducing stress to mice, in form of bleeding or bacterial infection-mimicking lipopolysaccharide (LPS), triggers multiplication of endosteal OCs. Furthermore, OC enzymes like MMP-9 and cathepsin K were present in increased quantities in the endosteal niche, loosening HSC anchorage. This released them into blood vessels (78), thus opposing blood loss and ensuring O_2_ supply. Contrary to these findings, studies have shown that OC inhibition did not influence HSC mobilization by G-CSF in mice (79), suggesting that OC influence on HSC mobilization is dependent on multiple factors. OCs may instead regulate HSC development indirectly by releasing Ca^2+^ and cytokines (TGF-β) into HSC cavities while resorbing bone mass (80, 81).

Recent studies from Grüneboom et al. have revealed an abundance of microscopic canals spanning from the bone marrow to the endosteal face of mice femurs, coining them transcortical vessels (TCV). These TCVs, aside from contributing immensely to general blood flow of the bone, were shown to be utilized by (neutrophil) granulocytes to emigrate from the bone marrow to extraosseal blood vessels when the host was injected with G-CSF—even against the direction of blood flow. TCV formation is dependent on OC activation, as blockade of OCs (with zoledronate) resulted in highly decreased TCV number within 4 weeks. Furthermore, Grüneboom et al. portrayed a significant increase of TCVs in chronic, but not acute, arthritis. Interestingly, these findings do not transfer to non-bone-related inflammation (82). New extraction methods have made it easier to gain OCs from peripheral blood with minimal effort and strain (83), making research on these cells significantly less elaborate.

## Main Components of the Immune System Viewed in Relation to Osseous Development

The immune system is an extremely diverse and powerful defensive tool of higher organisms. Development of the innate immune system began in unicellular, amoebic organisms. In fact, these amoebic life forms closely resemble macrophages as our immune system contains them today (84). Takayanagi et al. posit multiple reasons why the immune system is mainly housed inside the skeletal system. First, bone tissue shields stem and progenitor cells from harmful UV light, possibly damaging precious DNA and causing catastrophic replication errors. Secondly, the transition from aquatic to terrene environments brought along significantly higher concentration of oxygen, another possible danger to DNA. Ultimately, decreasing levels of external calcium (sea water contains about 400 mg/l of calcium) might have driven immunopoiesis to the bone marrow, where large quantities of calcium could be released quickly if needed, preceding the assumption, that calcium plays a crucial role for hematopoiesis (80, 85).

The innate branch of the immune system covers components, which are able to repel intruders on contact without earlier programming or further modification. It is extremely fast, identifying and attacking parasites within seconds of invasion, and due to its non-specific nature highly versatile (6).

In contrast to the innate immune system, the adaptive immune system requires multiple contacts with an antigen in order to develop the full strength of its response. First contact with an antigen is followed by programming to react specifically to this specific antigen. This makes the adaptive immune system slower to react than the innate immune system, but it compensates by its exquisite precision and, brilliantly, gives the ability to form an “immunological memory,” storing prior antigens for any number of years. Only this memory made it possible for modern medicine, starting with Edward Jenner, to actively immunize the public.

The immune system's aforementioned diversity stems from it being comprised of various cells, all different in form and function. Despite its diversity, all cells of the immune system descend from the HSC niches in the bone marrow, which are tended to all cells of the osseous line, mesenchymal cells and endosteal cells (86). Depending on developmental factors secreted from these local cells, the stem and progenitor cells commit to different lines of evolution (Table 1) (85).

**Table 1:** Important factors for hematopoiesis/immunopoiesis.

**Cytokine**	**Function/Target**
M-CSF	Macrophage development
G-CSF	Granulocyte development
GM-CSF	Macrophages, granulocytes
IL-2	T cell proliferation
IL-3	Possibly stimulation of the complete range of blood cells
IL-4	Cofactor
IL-5	Differentiation of b cells, eosinophil regulation
IL-6	B cell Differentiation
IL-7	T Lymphocyte formation
IL-11	Megakaryocytopoiesis
LIF	Leukemia inhibiting factor
SCF	Stem cell factor, proliferation of stem cells
FLT3	Proliferation of stem cells
TPO	Thrombopoietin, Platelet production

### Granulocytes

Granulocytes are divided into three groups according to their responses to different dyes.

#### Neutrophil Granulocytes

Neutrophil granulocytes (NG) are one of the fastest cell types to respond to invading bacteria, have a short lifespan of about 3 days and mostly reside in the interstitium of organs. The mechanisms of defense include secretion of multiple factors upon contact, such as myeloperoxidase (part of the oxidative burst, to destroy bacterial membranes) and lysosomal enzymes. In addition to these properties, NGs break down, and phagocyte remains of dead cells (87).

NGs are present in great numbers in early fracture and hematoma. When treated with anti-NG antibody 24 h before artificially inflicted fractures, mice have shown elevated concentrations of IL-10 and IL-6, as well as other pro- and anti-inflammatory cytokines and chemokines. Additionally, the fracture site contained significantly more monocytes/macrophages and presented an overall impaired healing process (88), suggesting that NGs play an important role in conducting chemo- and cytokines in inflammatory response.

Furthermore, when injected with G-CSF, a key regulator of NGs, pre- and postoperatively, rats profited from increased femur stiffness 5 weeks after mid-femur osteotomy in comparison to a placebo-control group, indicating a positive effect of G-CSF on early fracture healing (89).

#### Eosinophil and Basophil Granulocytes

Both of these types of granulocytes have not yet been correlated to the topic of osteoimmunology. Although their contributions to the immune system are indispensable, they are, as of now, negligible in this topic. It is, however, to be pointed out that mast cells play a fundamental role in fracture healing and osteoclast activation through the production of histamine, a trait shared with basophil granulocytes (90). These findings suggest, that basophil granulocytes may also play a role in these matters.

#### Monocytes/Macrophages

Monocytes (MC) are the progenitor cells of macrophages (MP) and dendritic cells. After circulating for about 1 d, monocytes leave the bloodstream to mature into macrophages in extravascular tissue (6). Both, monocytes and macrophages, are competent in phagocytosis and do so either with or without the help of antibodies. MCs produce IL-12, TNFα, and iNOS after contact with microbial antigens (91).

MPs are smaller than MCs and patrol the tissue of every organ of the human body. As their main function is phagocytosis, they react to wreckage of dead cells as well as to exogenous pathogens. Upon contact with pathogens, MPs release chemokines to attract other cells of the immune system, and after phagocytosis will present structures from devoured cells to lymphocytes amongst others (antigen presentation).

Due to their shared heritage and structural similarities, osteoclasts have often been described as the osseous representation of macrophages. While this is certainly correct, there are in fact other macrophages, which are characteristic for osseous tissue: namely bone marrow macrophages and osteal macrophages (osteomacs). Of course, their purpose is also defense against invading pathogens, but both subtypes of macrophages fulfill specific functions regarding bone turnover and stem cell keeping (92).

Osteomacs closely cooperate with megakaryocytes, progenitor cells of later blood platelets, and osteoblasts to maintain HSC niches. Osteoblasts work most effectively, regarding HSC keeping, when supported by megakaryocytes and osteomacs (93). *In vitro* it has been shown, that osteomacs can be triggered into becoming osteoclasts by stimulation with RANKL and M-CSF; *in vivo* this effect plays a relatively small role in osteoclast synthesis, suggesting that this process is based almost exclusively on development from HSCs (93, 94). As bone marrow and active bone formation sites are right next to each other and share multiple cytokines, osteomacs need to form a phagocytic barrier between the two, to stop signaling molecules from unintentional “roaming” (95).

Bone marrow macrophages can be further categorized into erythroblastic island macrophages (EIM) and HSC niche macrophages (HNM) (95). EIMs, as their name suggests, are closely linked to erythropoiesis. *In vivo* depletion of EIMs resulted in loss of all erythroblasts and reticulocytes in BM, while erythroblast precursors remain unaffected. The absence of anemia in EIM-depleted mice suggests shift of erythropoiesis to extramedullar tissue and modification of erythrocyte-sorting (96). HNMs serve a broader spectrum of HSCs, while controlling self-renewal and decommission of HSC. Recent studies by Vinchi et al. have pointed out the potential of HNM manipulation for bone marrow transplantation (97).

Evidence emerges that a subpopulation of MPs does not descend from blood-circulating MCs but instead occupy organs as early as embryonic development and self-sustain independent of MCs (98, 99). *Nature* recently published an article detailing the origin and function of MPs in organs and particularly in joints. CX_3_CR1^+^ positive, tissue resident MPs form a layer at the synovial coating, not unlike an epithelial layer, providing an immunological barrier. These MPs are locally renewing, independent of circulating recruitment and differ from illuviated MPs by restricting inflammation through tight junctions (100), instead of fostering it. Oppression of non CX_3_CR1^+^ positive MPs while simultaneously sustaining CX_3_CR1^+^ positive MPs might be beneficial for inflammative joint diseases.

### Lymphocytes

Lymphocytes are the main representatives of the adaptive Immune system and comprise of B and T cells. Both these subsets derive from the same lymphatic progenitor cell during hematopoiesis, and while B cells mature in the bone marrow, T cells complete their maturation process in the thymus. B cells as well as T cells need to fulfill certain conditions to be deemed immune competent: (1) Recognition and ability to bind to extraneous antigens with their respective membranous AG receptors, while non-binding to endogenous AG (self-tolerance); (2) Working sets of CD4/CD8 co-receptors; (3) Ability to bind to presented AG by different cells. All cells not capable of achieving any of these conditions are sorted out and phagocytized by local macrophages (6).

### B Cells

B cells are responsible for incorporating the humoral component of immune response by turning into plasma cells when triggered and producing specific antibodies. B cell development is dependent on RANKL, CXCL12, and IL-7 exposition in the HSC (101), with studies proving the dependence on RANKL by raising RANK^−/−^ mice, which showed normal MALT (mucosa-associated lymphatic) tissue development, but suffered from a complete absence of peripheral lymph nodes (102). B cells themselves produce RANKL to stimulate B cell predecessor maturation in an autocrine fashion (99), but supposedly are not in need of RANK as receptor for RANKL to do so (103).

The aforementioned IL-7 stems from OBs of the HSC niche and is dependent of mTORC1 messaging. By deactivation of the mTOR complex in OBs, B cells were significantly reduced in the bone marrow, highlighting the importance of OBs for B cell maturation (104).

Interestingly, very early B cell progenitors, when treated with ODF/RANKL and M-CSF *in vitro*, could potentially turn into fully functioning osteoclasts, questioning whether the lymphoid development might be reversable (105). These findings, however, cannot as yet be reproduced *in vivo* (106). Memory B cells have an immense capacity in production of RANKL and actively stimulate OC maturation. Especially in RA patients, memory B cells are overly active with RANKL production and likely contribute to joint destruction (66). B cells also contribute to osteoporosis in splenectomised rats with higher B cell count in the bone marrow (107). B cells inhibit osteoblastogenesis through notch signaling, a pathway that modifies OBs and OCs positively or negatively depending on context (108). Taken together, these studies emphasize a rather catabolic function of B cells regarding bone homeostasis.

### T Cells

T cells are categorized by distinction of their cell surface molecules (CD = Cluster of Differentiation). CD4^+^ T cells are called T helper cells, further divided into Th1, Th2, Th17, and regulatory T cells (T_reg_). These subsets differ in produced cytokines and therefore function (109).

Especially Th17 cells have great influence on bone metabolism by releasing IL-17A, IL-17F, IL-22, and IL-26 when activated in conjunction with TGF-β and other inflammatory factors. IL-17A results in activation of NFκB, increasing quantity and productivity of osteoclasts (110, 111). Th17 cells are also capable of RANKL production independently, but not as much as to directly promote osteoclastogenesis (112) and, most interestingly, upregulate RANK exhibition on osteoclast precursors (113).

In the event of bacterial infection of bone tissue, Th17 and Th1 cooperate to limit spread of infection and do so by supporting bone resorption (RANKL↑) (114). Contradictory to these findings, it has been shown, however, that both Th1 and Th2 inhibit osteoclast formation by secreting IFN-γ and IL-4 (115). These studies make it obvious that T helper function greatly depends on context, namely physiological or pathological conditions.

T_reg_ cells, on the other hand, have a relatively strict anti-osteoclastogenesis function. In rheumatic patients the number of Foxp3^+^ T_reg_ cells is inversely related to osteoclastogenic markers. These results accompany findings of the same group, in which T_reg_-deficient mice were prone to arthritis, but reintroduction of T_reg_ into these mice via bone marrow transfer significantly reduced symptoms such as decreased paw grip strength, weight loss and paw swelling. When compared to wild type mice, Foxp3^+^ T_reg_-deficient mice show greater histological TNFα-induced joint destruction and generalized bone loss with higher number of osteoclasts in joints (116).

CD8^+^ T cells, or cytotoxic T cells, induce apoptosis in targeted cells by releasing perforin and granzymes and activation of FAS receptors (117). In 2017, Savola et al. examined patients with newly diagnosed RA and found somatic mutations of CD8^+^ T cells in 20% of patients while a healthy control group showed only 5% with said mutation. Some of these genes were linked to autoimmunity beforehand and are believed to have effects on protein production, indicating possible involvement of CD8^+^ cytotoxic T cells in RA (118). Other studies have pointed out the involvement of CD8^+^ T cells in signal transduction of PTH, increasing pro-osteoblastic, pro-bone growth cytokines of the Wnt10 class when triggered by PTH (119).

Information on the involvement of cytotoxic T cells in bone degeneration is sparse and contradictory and should be further suspect of investigation. Characterizing factors of T cells are summarized in Table 2 (70, 120).

**Table 2:** T cell subsets, differentiating factors and function.

**Cell**	**Development factors**	**Exhibited factors**	**Function/target**
Th1	IL-12	IFN-γ, IL-2, LTα, IL-10	Intracell pathogens
Th2	IL-2 + IL-4	IL-4, IL-5, IL-10, IL-13, IL-25	Parasites, allergy
Th17	IL-6, IL-21, IL-23	IL-10, IL-17A, IL-17F, IL-21, IL-22	Bacteria, fungi
T_reg_	IL-2	IL-10, IL-35, TGF-β	Immune tolerance, regulation of immune response

*Development factors describe agents needed for T cell differentiation and are provided by local cells or systemically*.

### Bone Turnover

Physiological bone turnover is a lifelong process and includes both bone resorption and bone synthetization. Structural changes in aging individuals, such as decrease of bone mass, osteophytes and joint alterations, are somewhat physiological processes (121) constituting the shift or reduction of cellular activity. Only when these changes precede the actual age of any individual is it to be considered pathological.

Generally speaking, it is crucial to differentiate between bone modeling and bone remodeling. Bone modeling is characterized by gain of mass. Although both, OCs and OBs, are active in this process, synthesis of new matrix outweighs osteolysis. It can be observed either in adulting (growing) individuals or after bone fractures (122). Fracture healing is of particularly high interest in the field of osteoimmunology because it involves large contributions of the immune system.

#### Fracture Healing

Shortly after, or in the process of fracture, blood vessels surrounding the bone rupture und release blood into the injury site. This forms the hematoma and allows immune cells, neutrophils, macrophages and lymphocytes to infiltrate the tissue and release a multitude of growth factors and cytokines (123, 124).

Macrophages are among the quickest cells to respond and contribute to healing throughout the whole process of modeling. Macrophages' main task in the inflammatory phase of fracture healing is clearance of debris and defense against possible pathogens (125, 126). Two different types of macrophages have been identified, M1 macrophages, which primarily react to infection with various organisms, and M2 macrophages, which are preferentially involved in tissue regeneration. Both types play a role in bone fracture healing, being attracted by the expression of CXCL12 of damaged tissue, a process that is enhanced by TNFα (126). In fracture healing, TNFα from macrophages also sensitizes OB progenitors to growth factors and enhances OB differentiation (in combination with IL-6) (19). Toward the end of the inflammatory phase, activated immune cells secrete factors to attract and stimulate mesenchymal progenitor cells (127), which in turn limit inflammatory activity (128).

During the phase of cartilage formation, mesenchymal progenitor cells evolve into chondroblasts, rather than OBs, triggered by mechanical instability and chemical attractants (123). These cells undergo apoptosis under the influence of TNFα (129); this happens once the amount of synthesized cartilage is sufficient for stabilization and then vascularization ensues. This still comparatively soft tissue is then mineralized and used by osteoprogenitor cells as a framework for the deployment of rigid, osseous tissue (122, 123, 129) and is eventually removed (124, 130).

The last phase of fracture healing is marked by transformation (meaning resorption and synthetization) of the woven bone into laminar bone, finally reconstituting the former matrix constellation (123).

To clarify the importance of immune cells for bone fracture healing, Vi and Baht demonstrated that macrophage depletion in adult mice delays bone fracture healing, while macrophage depletion in developing mice resulted in osteoporosis and growth retardation, which points out macrophages' importance in bone modeling as well as remodeling (131). However, in contrast to these findings, Ono and Takayanagi have shown that TNFα also inhibits bone mineralization (19). Further investigation is necessary to clarify the explicit effects of TNFα onto the osseous cells. The dichotomy of these findings makes it seem possible that TNFα promotes quick bone turnover, albeit at the expense of matrix quality.

T helper (Th) cells of the lymphoid lineage, specifically Th17, promote osteoblast maturation with the secretion of IL-17F (132), while B cells are capable of OPG production to ensure OC supervision (133). Table 3 summarizes contributions of the immune system to bone fracture healing.

**Table 3:**
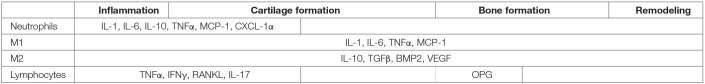
Secreted factors and relevant immune cells in bone fracture healing.

*Macrophages (M1 and M2) are active throughout the complete healing process, with M1 stimulating osteoclasts and M2 stimulating bone formation from OBs. Lymphocytes shift from inflammatory modus to secretion of OPGs when bone formation is required*.

Due to the inflammatory nature of bone fractures, it has long been obvious that components of the immune system play a vital role in the healing process. Recent studies have enabled a glimpse of the extent of intracellular cooperation necessary, constantly identifying new actors, yet still large portions remain to be uncovered. Fracture healing with a special focus on the influence of the immune system bears immense therapeutic possibilities and needs to be investigated further.

#### Bone Remodeling

Bone remodeling contributes to material exchange of osseous matrix and ideally results in an equilibrium of bone synthetization and adsorption. OBs and OCs work in a coordinated manner in both location and time as basic multicellular units (134). First OCs “drill” a channel into the compacta (a future osteon), or a lacuna into the cancellous bone (Howship-Lacuna) (135). On their way, previous osteon borders are disregarded and a new osteon forms, cutting into already existing ones (6). Following the OCs, OBs fill in the damage from the outside with new lamellae of matrix. While OBs and OCs handle the physical part of bone remodeling, osteocytes are the initiators, conducting both cell types from within their lacunae (not to be confused with Howship–Lacunae) using their arboreal network of dendrites (6).

The following stimuli are triggers for osteocytes to start the manipulation of osseous cells: (1) Shear force applied to the bone matrix is probably the most common signal—bone that is subjected to load will need remodeling to countervail material fatigue. Osteocytes will recognize movement of matrix fluids to measure the amount of strain (54). (2) PTH signals calcium need in the blood circulation, and result in a quick (~1 h) release of RANKL by osteocytes (136). In addition, osteocytes themselves have the ability to release calcium by dissolving their surrounding matrix through cathepsin K secretion (137). To keep the osteolytic process of OC activation in limits, PTH inhibits the synthesis of sclerostin, which, in turn, accelerates the Wnt pathway (see above), so PTH not only promotes osteolysis but also bone development. (3) Microdamage through excessive point loads requires rebuilding to ensure structural integrity of the matrix. Local damage leads to apoptosis of nearby osteocytes and subsequent upregulation of OC activity (138). To study the effects of osteocyte apoptosis on bone remodeling He and colleagues treated osteocytes with potentially destructive irradiation. Upon apoptosis, a significant increase of RANKL, decrease of OPG and thus a shift in RANKL/OPG axis was observed. Intracellular high mobility group box 1 (HMGB1), a pro-inflammatory and osteoclastogenetic protein, was increased, which contributes to osteolysis (139).

## Osteoimmunology in Total Hip Replacement

**Figure 4:**
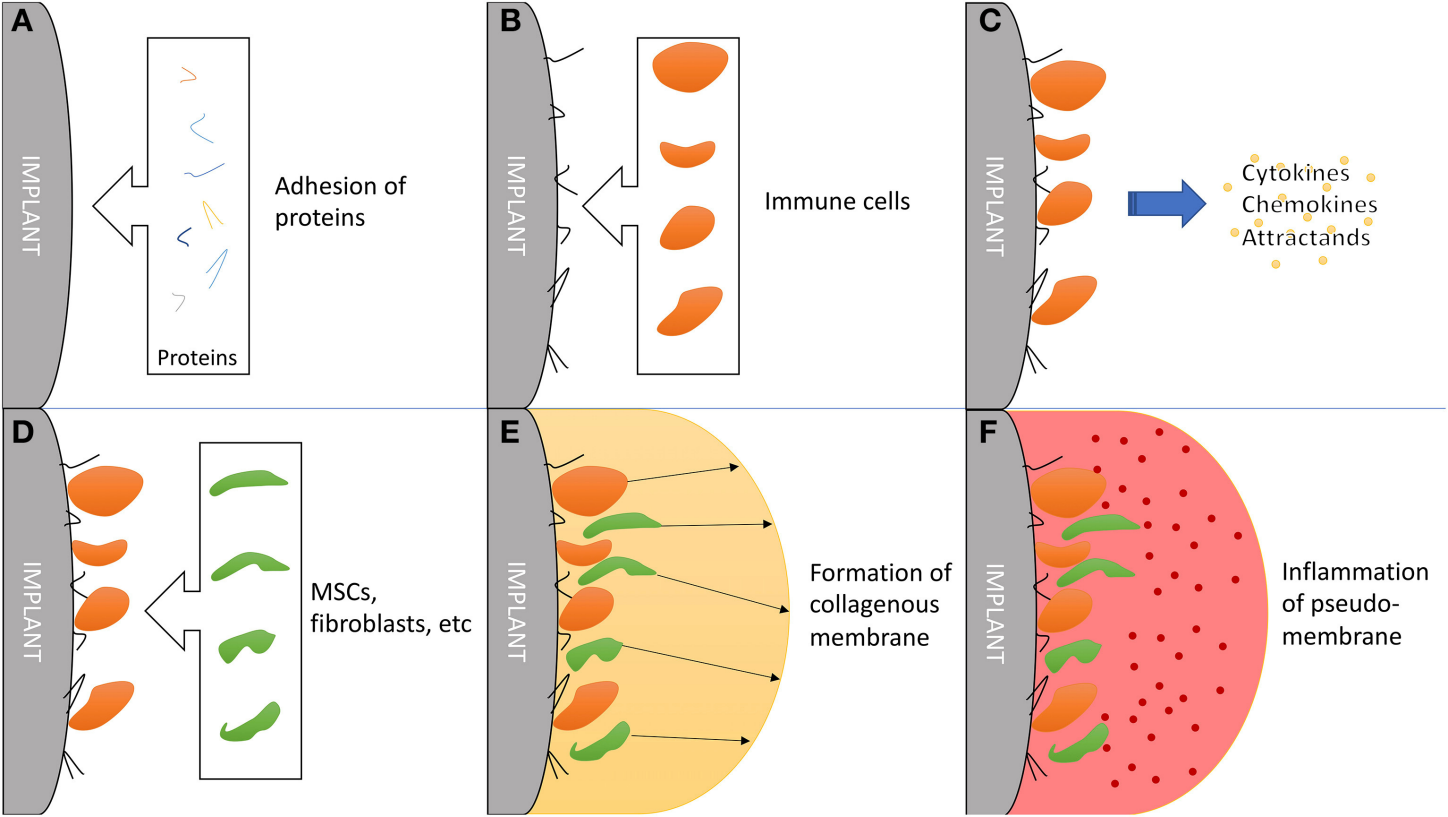
The critical role of the immune system in formation and function of a synovial-like periprosthetic membrane. **(A)** Adhesion of local proteins to prosthesis surface within minutes of surgery. **(B)** Attraction of blood cells: macrophages, monocytes and thrombocytes. **(C)** Secretion of mesenchymal (stem/stromal) cell attractants. **(D)** Homing of mesenchymal stem/stromal cells and fibroblast. **(E)** Production of collagen and granulomas to form a synovial-like capsule. This marks the last step in “physiological” prosthesis acceptance. **(F)** Mechanical instability and wear debris lead to chronic inflammation of the pseudo membrane. Inflammation markers are mainly exhibited by local fibroblasts and macrophages.

Hip implant loosening afflicts 3–10% of all patients within 10 years (140), posing an catastrophic event for patients with total hip arthroplasty (THA). Problematically, periprosthetic osteolysis, resulting in aseptic hip implant loosening, is asymptomatic for a long time, while the THA works as intended (141). Chronic inflammation resulting from microscopic abrasion from components has proven to be the most important factor, followed by initial, perisurgical instability (142, 143). The immune system plays a critical role in chronic inflammation (144) and additionally in formation and function of a synovial-like, periprosthetic membrane (Figure 4) (145).

Abrasions phagocytosed by macrophages result in the release of a cocktail of anti- and predominantly pro-inflammatory factors (146), and since component wear is continuous, chronic inflammation ensues. Pearle et al. confronted peripheral blood mononuclear cells and MCs with polymethylmethacrylate (PMMA) and measured released mediators and gene expression. The result was an up to 12-fold increase of pro-inflammatory cytokines (TNFα, IL-1α, IL-1β, IL-6, IL-8) and up to 30-fold increase of PTGS2 (COX2). OCs themselves reacted to confrontation with PMMA particles with upregulation and activation of NF-κB (147). Confrontation with titanium particles provoked pro-inflammatory response from T helper cells, with participation of IL-2, IL-9, IL-13, IL-22, and INF-γ (148). As previously discussed, multiplication of pro-inflammatory factors directly results in increase of RANKL expression and subsequent OC activation. Additionally, Atkins et al. found that OBs, when triggered by polyethylene particles, downregulate their production of OPGs, again resulting in a shift in the RANKL/OPG axis toward osteolysis (149). Wear particle size plays a strikingly significant role in inflammatory reaction. Debris with a size allowing phagocytosis might stimulate pro-osteolytic processes even before endocytosis, while larger fragments cause a similar, yet minor, reaction (150).

Regardless of stability of the prosthesis, a periprosthetic membrane forms shortly after the operation, with the membrane being thicker in cases of unstable mounting (151) and longer implant duration (140). The longer the prosthesis remains in the human body, the more inorganic debris from the prosthesis and the more active, local macrophages (histiocytes) can be found within the granulomatous membrane (151). The membrane promotes distribution of pro-inflammatory factors to surrounding tissue, and especially periarticular bone, through synthetization of moving synovial liquid (152). The synovial fluid of patients with loosened total hip arthroplasty contained significantly higher amounts of pro-osteolytic cytokines, like RANKL, IL-6, IL-8, and monocyte chemoattractant protein 1 (MCP1) than the synovia of unproblematic prosthesis (153).

From a cost perspective regarding joint revisions, total knee arthroplasty revisions alone posed a cost factor of $1.27 billion in the United States in 2005 (154), showing the relevance of minimizing negative osteoimmunological effects on total endoprosthesis. Both new prosthesis materials, as well as downregulation of overly active pro-inflammatory cells, need to be subject of further investigation. It should be kept in mind, that long-term immunosuppression cannot be considered a solution, because, as mentioned, material wear is constant and so the immune response would need to be suppressed from the moment of operation until removal of the prosthesis or death of the patient.

## Osteoimmunology in Rheumatoid Arthritis

A physiological, but not yet fully understood, process is the citrullination of proteins. Citrullination being the deamination of arginine to citrulline has not yet been proven to be reversible (155) and is carried out by enzymes of the peptidylarginine deiminase (PAD) group (156). Citrullination has been discussed as an early sign of cell damage as it occurs in many inflamed tissues, and, interestingly, even precedes detectable inflammation or disease (157, 158).

In patients suffering from rheumatoid arthritis (RA), the synovial fluid exhibits a unique pattern of citrullinated proteins, the RA citrullinome (159, 160), suggesting a dysregulation on genetic levels. Whether the dysregulation creates an abundance of previously tolerated, citrullinated proteins which then trigger an immune response due to mere mass, or results in proteins citrullinated in abnormal ways is still unclear (161). Either way, B cells produce antibodies against these citrullinated proteins, called anti-citrullinated peptide antibodies (ACPA) triggering a distinct immune reaction and successive inflammation and tissue destruction, marking one of the typical pathomechanisms of RA. These ACPAa are present in RA patients for many years before first symptoms (162, 163) and are strongly correlated with severity of some symptoms and are a useful prognostic assessment (164, 165).

**Figure 5:**
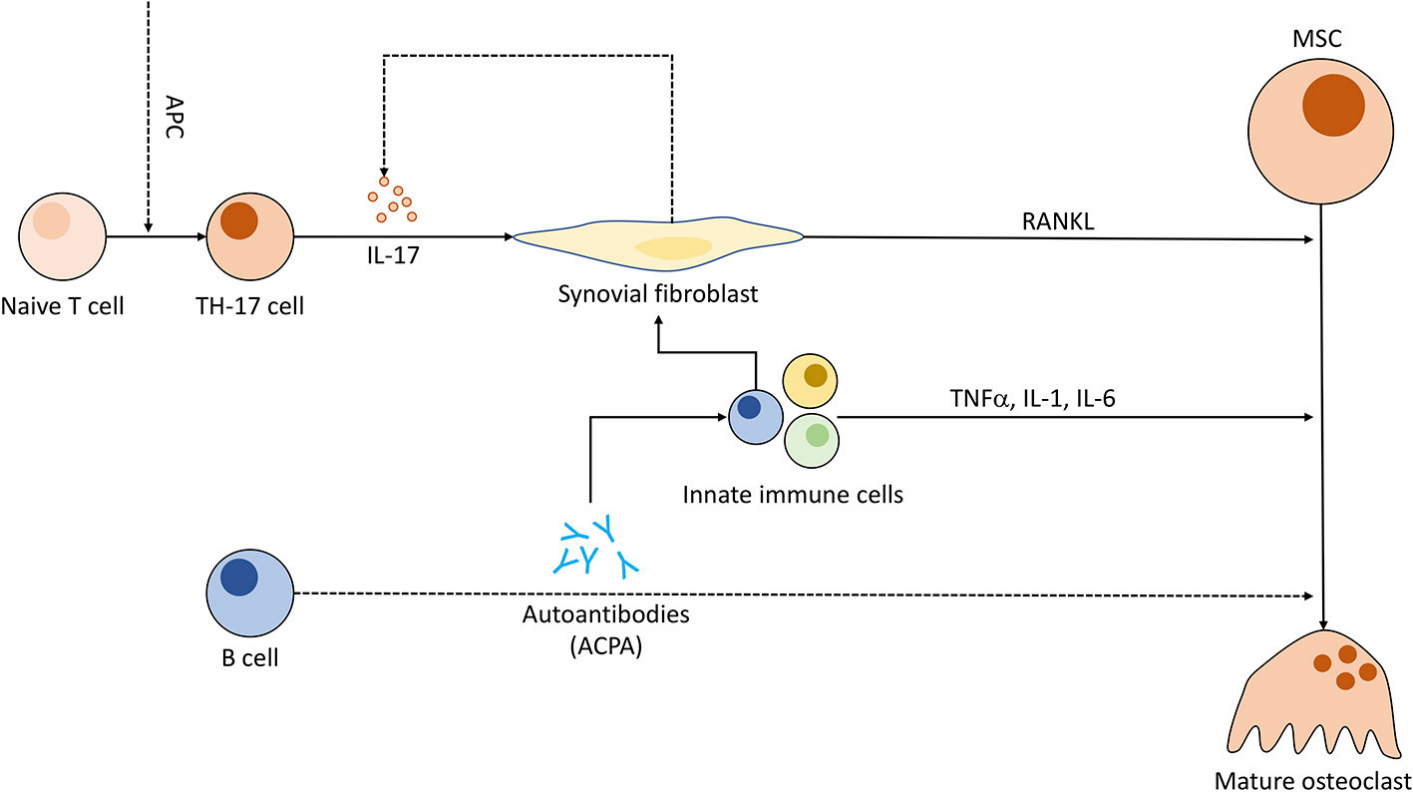
Pathogenic role of immune cells in rheumatoid arthritis and the connection to osteoclastogenesis. Antigen-presenting cells (APC) induce T cell differentiation to Th17 subgroup. Th17 cells, when triggered, emit IL-17, compelling fibroblasts to produce RANKL and indirectly stimulate increased IL-17 production. ACPAs (anti-citrullinated peptide antibodies) synthesized by B cells might be (dotted line) capable of enhancing OC differentiation and entice other pro-inflammatory immune cells to cytokine production. These immune cells in turn prompt fibroblasts to secrete even more RANKL.

Recent studies discuss the capability of ACPAs to bind directly to OC precursors, promoting osteoclastogenesis. A 2012 study has visualized binding of ACPAs to OCs *in vitro* and demonstrated bone loss in lymphocyte-deficient Rag1^−/−^ when injected with ACPAs against a control group (166). Other papers regarding this topic have been retracted or corrected (167–169) due to errors in methodology and further information remains sparse. Research regarding ACPA-specific implications on OCs seems worthwhile to the advancement of RA treatment. Nevertheless, IL-6, a cytokine otherwise associated with activation of granulocytes and pro-angiogenetic effects (170), has been related to ACPA-induced bone loss and its inhibition has shown great potential for RA treatment (Figure 5) (171).

Recently Chen et al. published an article characterizing anti-inflammatory and de-escalating cytokines. While it is true, that cytokines play an integral role in the onset and development of RA, they also have the ability to moderate and dampen inflammatory processes. Production of IL-4 and IL-13 by Th2 cells triggers macrophage transition from type M1 to M2, which in turn release IL-10 and TGF-β, effectively preventing macrophage and neutrophil infiltration into joints and reducing pro-inflammatory cytokine production (172). IL-9, too, is competent in reducing negative effects of RA by forcing differentiation of T_reg_ cells (173).

Still, definitive statements about cytokine functions are to be viewed cautiously, since many (if not all) cytokines have context-dependent tasks. IL-33, for example, is generally considered a pro-inflammatory cytokine, capable of provoking immune cell migration and mast cell activation (174). However, in the later stages of inflammation, IL-33 serves limiting purposes (175, 176). Examples like these highlight the importance of further investigation on the topic of cytokines, investigating context dependent functions and contemplating possible treatment options.

## Osteoimmunology in Osteoporosis

Simply put, osteoporosis is an imbalance between bone destruction and development, with OC surpassing OB in activity (177). The result is loss of absolute bone mass and fragility of remaining osseous tissue (178), which is most common in postmenopausal women (179). This fact leads to the assumption and subsequent conclusion that estrogen plays a key role in the pathology of osteoporosis (180).

While estrogen has great influence on cells of the osseous lineage, e.g., assuring osteocyte survival (181–183), preventing OB apoptosis and increasing their lifespan by triggering Sema3A secretion in osteocytes (184, 185), downregulating OC activity and lifespan (186, 187) and inhibiting RANKL effects on OC (inter alia by upregulating OPG production in OBs) (188–192), estrogen also affects bone metabolism indirectly by stimulating immune cells. Estrogen has been shown to downregulate RANKL production in lymphocytes (177) and modulate production of inflammatory cytokines which eventually cause bone resorption in osteoporosis as well as RA (193). IL-6 production is inhibited on a transcriptional level by binding to a cellular receptor on producing cells (194). IL-6, in fact, enhances both OB and OC activity (bone turnover ↑), but OC activity surpasses, causing bone loss over time (195).

Furthermore, blocking of IL-1 and TNFα receptors simultaneously completely negates bone loss after ovariectomy, indicating that both factors are highly relevant for estrogen-related bone loss (196–198). Effects of estrogen on TNFα levels is indirectly meditated by T cells (199), demonstrating the importance of T cells for estrogen-mediated bone preservation. Even further, Cenci et al. found that T cell-deficient mice, when ovariectomized, show no signs of increased osteoclast formation, while WT mice exhibit twice as many osteoclasts (200). These results have been questioned by findings of two individual groups, which demonstrated that mice which lost T cell function by different methods, still show the same amount of cancellous bone loss after ovariectomy (201, 202).

**Figure 6:**
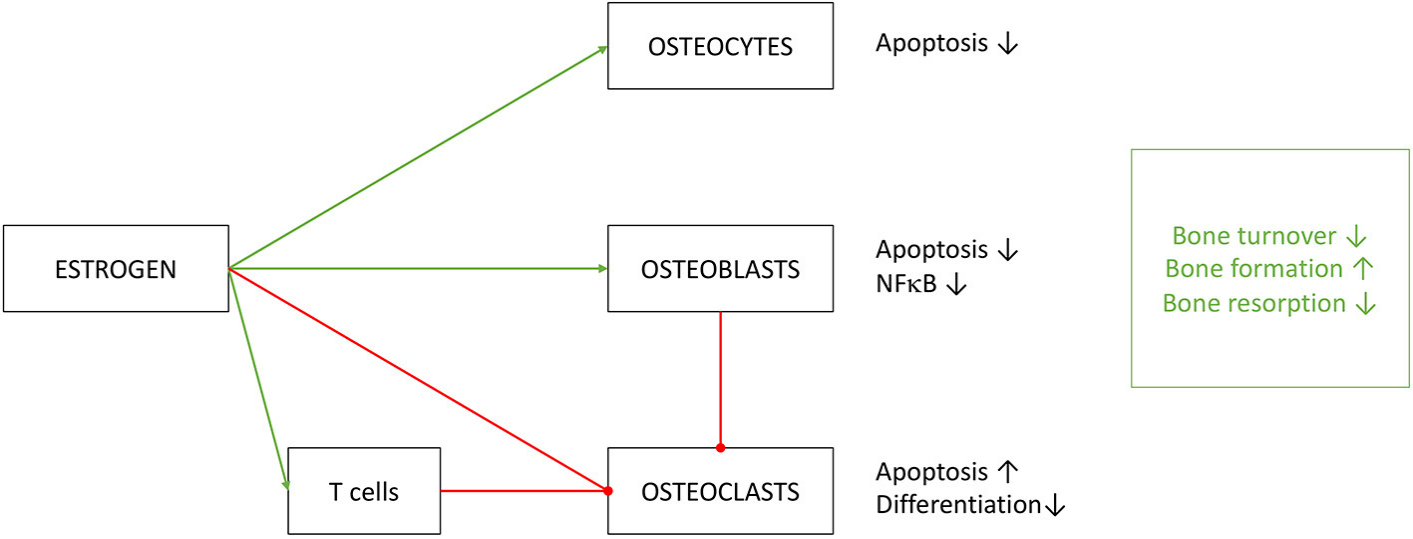
Effects of estrogen on cells of osseous heritage. The simplified presentation highlights inhibitory (red) effects on osteoclasts and promoting (green) effects on osteoblast and osteocytes. Estrogen effects are in part executed by T cells, especially on OC metabolism. Results of estrogen interference are enhanced bone formation with decreased turnover and resorption.

To summarize, estrogen clearly has a significant effect on bone turnover and does so by the extensive help of the immune system (Figure 6) (203). Regulating key effectors of the immune system, like IL-1, IL-6, TNFα and prostaglandins may be beneficiary for osteoporosis therapy. Contributions of the immune system to osteoporosis are so distinct that Srivastava et al. introduced the term *immunoporosis* in 2018 (70).

## Osteoimmunology in Heterotopic Ossification

Heterotopic ossification (HO) depicts the phenomenon of pathological osseous growth in consequence of trauma, large surface area burns, surgery, spinal cord injury and traumatic brain injury. In HO, physiological constitution of functional or scar tissue fails and instead is replaced by the formation of ectopic osseous tissue (204).

Origin of HO is still largely unknown, although severe inflammatory responses (both locally and systemically) and wound infections have been found to aggravate the condition (205). In case of HO caused by military blast wounds, research has detected a high concentration of inter alia TGF-β, platelet, epidermal and fibroblast growth factors, IGF-II, prostaglandins, TNFα (206) and, most notably, BMP-4 (207, 208). This inflammatory compound exerts osseous formation from circulating and local osteogenic precursor cells (208, 209) supported by the hyperactive immune system and other surrounding tissue.

HO forms and solidifies within ~6 months to 1.5 years after initiation and is accompanied with all effects of tumorous growth: Pain, restricted range of motion, pressure ulcers and complications due to compression of adjacent structures like vessels, nerves, and muscle (210, 211). Surgical excision is the ultimate treatment of HO, but not always possible due to proximity to sensitive structures or patient's status, and bears the risk of recurrence if not removed completely (212). If possible, e.g., for operations with high chance of HO formation, prophylactic measures can be taken and currently comprise of radiation therapy (213), NSAIDs (most prominently indomethacin) (214) and, rarely, corticosteroids (215).

Since more and more immunological players and messenger substances have been identified to contribute to HO, the focus of research has shifted toward osteoimmunology. A 2014 study has utilized transgenic mouse models of HO with overexpression of BMP4 to prove that depletion of macrophages reduces HO. Introducing tissue injury to a mouse with BMP4 overexpression resulted in HO development within 4 weeks. When depleted of macrophages, however, these mice were less likely to develop HO, showing the importance of macrophages for HO formation. Furthermore, this study suggested, that spreading of HO outside of initial injury was mediated by the adaptive immune system (216). As representatives of the adaptive immune system, lymphocytes show close proximity to herds of HO (217) in cardinal valve ossification. Other than macrophages, mast cells of the innate immune system have been linked to the initiation of HO. Mouse models with attenuated activity of mast cells are less prone to HO (218, 219) due to changes in concentration of inflammatory peptides like CGRP and substance P.

Fibrodysplasia ossificans progressiva (FOP), a rare genetic disease, causing formation of bone in soft tissue, shows very close resemblance to HO and some treatment options for both conditions have been proven to be interchangeable. In 2015, Hatsell et al. and Hino et al. demonstrated that the mutation of the ACVR1 gene caused aberrant activation of the Activin A Typ 1 Receptor by activin A ligand resulting in upregulation of the pSMAD1/5/8 pathway and thus formation of osseous tissue (220, 221). Further testing of these finding *in vivo* yielded creation of an anti-activin A antibody (REGN2477) which is currently examined in a phase 2 study (registry NCT03188666 on Clinicaltrials.gov).

In summary, the involvement of immune cells and substances of the immune system make heterotopic ossification an obvious target for therapeutic intervention. As stated before, common therapy includes NSAIDs as classical immunosuppresiva, implying all risks and side effects. Biologicals, targeting specific mediators of HO locally should prove to be a far less invasive treatment option.

## Future Prospects of Applied Osteoimmunology

Immunology has become a major part of many different medical fields; unfortunately, the connection of immunology to orthopedics and trauma surgery has been developing rather slowly. Discoveries in recent years have emphasized the immense interplay between both systems and the arising therapeutic opportunities.

For cancer treatment, monoclonal antibody therapy has marked a milestone, significantly improving survival rates and recidivism-free years. This strategy has been integrated into the treatment of rheumatoid arthritis with high success as well: notably Tocilizumab (Target: IL-6R), Adalimumab (Target: TNFα) and Goliumab (Target: TNFα) are approved and highly successful therapeutics as mono- or combination therapy with DMARDs (222–227). Further investigation for possible targets of monoclonal antibodies is mandatory. As mentioned, ACPAs play an important role in development and upkeep of ACPA^+^-RA but are yet to become more than a prognostic marker. An ACPA antibody (antibody-antibodies) could limit the vicious circle of antibody generation and inflammatory reaction even before the onset of clinical symptoms.

**Figure 7:**
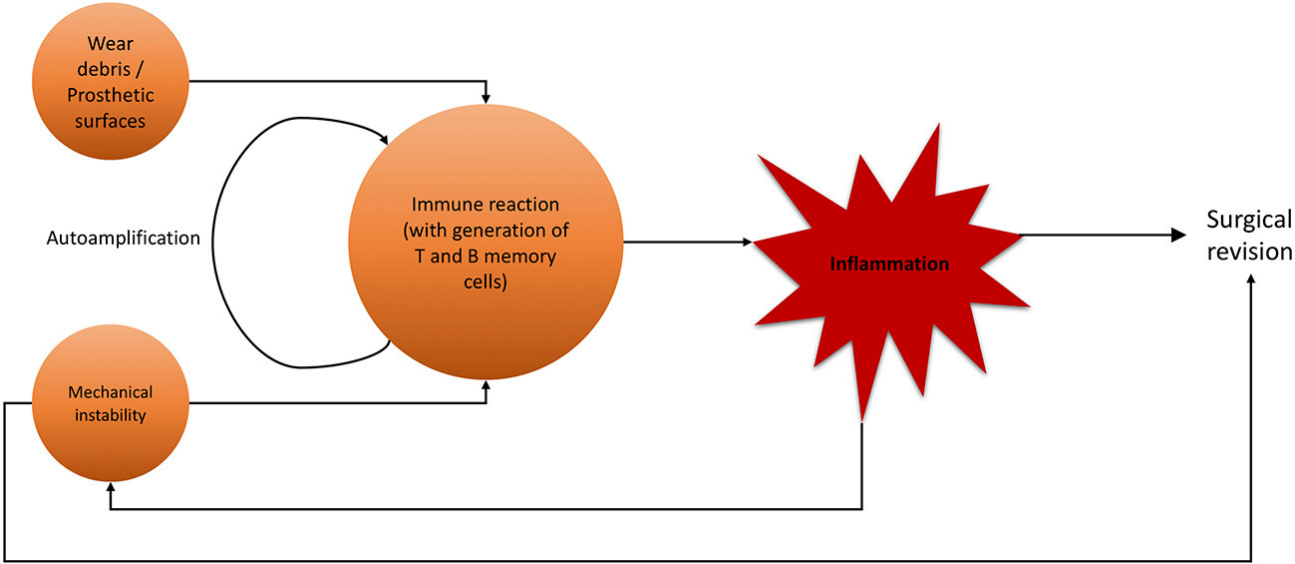
Flowchart of noxae promoting aseptic hip implant loosening and subsequent surgical revision. Wear debris and mechanical instability are major agents for inflammatory immune reaction, not limited to lymphocytes.

For aseptic (hip) implant loosening, several targets for conservative therapeutic actions are possible (Figure 7). As previously stated, there is a definitive immune reaction to wear particles with phagocytosis and/or direct inflammatory response. Recent efforts have been made to not only create non-immunogenic materials, but also to give immune-modifying properties to employed materials. While no material can be guaranteed to never trigger an immune response, it is possible to fabricate biomaterials with beneficial effects on the immune system. By modifying the surface qualities of biomaterials, it is possible to provoke a favorable, non-degenerative, reaction of macrophages (228). This marks a crucial step up from efforts to exclude the immune system from implantation to actively incorporating and benefiting from it. Researching these materials, giving them supportive immune-modulating effects while maintaining stability and longevity is crucial for future orthopedics and trauma surgery.

It might be beneficial to include general standardized allergy testing regarding metals and inorganic materials as a pre-op measure. Although the inflammatory reaction to wear debris and prosthetic surfaces are most likely caused by constant triggering of the immune system over long periods of time, rather than *conventional* allergic reactions, some patients *do* react to the materials in a type 4 allergic reaction. For these patients, careful selection of materials used is vital to prevent unnecessary revision procedures. A retrospective study by Zondervan et al. in 2019 presented patients with all-around improved testing scores (pain, walking quality, Range of motion) when revised to a hypoallergic component in total knee arthroplasty (229). Some of these patients may have avoided a secondary operation if tested beforehand and treated accordingly.

Regarding osteoporosis in postmenopausal women, osteoimmunology might be able to convey the key to slowing and/or stopping progression of osteolysis. Considering that the effect of estrogen-deficiency is partly T cell and MC-dependent (230), it might be possible to modify immune activity in a beneficial matter. To do so, the dichotomous effects of estrogen on estrogen receptors (ERα and ERβ) of immune cells need to be unraveled, so that absence of estrogen stimulation can be compensated. Again, long-term immunosuppression or intensive hormone therapy is not a preferable therapy for its excessive and unfathomable consequences for human physiology. Instead a very specific stimulation of immune-related ERα/β could be a solution.

These considerations are exemplary for the developing field of osteoimmunology. Moving away from systemic application (=immunosuppression), toward highly specific and acute modulation of the immune system. Unraveling conjoined mechanisms of the immune system and bone offers therapeutic possibilities for ailments of both systems. Contemplation of the bone as merely an unchanging support pillar is outdated and obsolete. Instead, it is mandatory that this extremely varying network is incorporated in our view on the immune system and hematopoiesis.

## Author Contributions

All authors contributed to conception and design of the study. CG and FS researched and wrote the content of the review article. All authors contributed to manuscript revision, read, and approved the submitted version.

### Conflict of Interest

The authors declare that the research was conducted in the absence of any commercial or financial relationships that could be construed as a potential conflict of interest.
